# Treatment with the selective serotonin reuptake inhibitor, fluoxetine, attenuates the fish hypoxia response

**DOI:** 10.1038/srep31148

**Published:** 2016-08-08

**Authors:** Jennifer M. Panlilio, Sara Marin, Marissa B. Lobl, M. Danielle McDonald

**Affiliations:** 1 Department of Marine Biology and Ecology, Rosenstiel School of Marine and Atmospheric Science, University of Miami, Miami, FL 33149, USA; 2 Department of Biology, University of Miami, Coral Gables, FL 33146, USA

## Abstract

The selective serotonin reuptake inhibitor (SSRI) fluoxetine (FLX), the active ingredient of the antidepressant drug Prozac, inhibits reuptake of the neurotransmitter, serotonin (5-HT; 5-hydroxytryptamine), into cells by the 5-HT transporter (SERT). Given the role of 5-HT in oxygen detection and the cardiovascular and ventilatory responses of fish to hypoxia, we hypothesized that treatment of the Gulf toadfish, *Opsanus beta*, with FLX would interfere with their response to hypoxia. Toadfish treated intra-arterially with 3.4 μg.g^−1^ FLX under normoxic conditions displayed a transient tachycardia and a biphasic caudal arterial blood pressure (P_CA_) response that are in direct conflict with the typical hypoxia response. Fish injected intraperitoneally with FLX under normoxia had resting cardiovascular and ventilatory parameters similar to controls. Upon exposure to hypoxia, control toadfish exhibit a significant bradycardia, reduction in P_CA_ and an increase in ventilatory amplitude (V_AMP_) without any changes in ventilatory frequency (fV). Fish treated IP with 10 μg.g^−1^ FLX showed an interference in the cardiovascular and ventilatory response to hypoxia. Interestingly, when treated with 25 μg.g^−1^ FLX, the bradycardia and V_AMP_ response to hypoxia were similar to control fish while the P_CA_ response to hypoxia was further inhibited. These results suggest that SERT inhibition by FLX may hinder survival in hypoxia.

Fluoxetine (FLX), the active ingredient in the antidepressant, Prozac (Eli Lilly and Company), is among the most highly prescribed selective serotonin reuptake inhibitors (SSRIs)^1^. It prevents cellular reuptake of the monoamine neurotransmitter, serotonin (5-hydroxytryptamine; 5-HT) by the 5-HT transporter (SERT), resulting in an increase in extracellular 5-HT that can lead to a rise in circulating 5-HT concentrations^2^,^3^. After consumption, FLX is metabolized and then excreted from the human body primarily through the urine with up to 11% of the administered FLX dose being excreted as the unchanged parent compound^4^,^5^,^6^. The excreted FLX and its potent metabolite, norfluoxetine, which also acts at SERT, then enter wastewater treatment facilities but are not effectively removed^5^,^6^,^7^,^8^. As a result, both compounds are detectable in aquatic ecosystems, especially in areas with major municipal wastewater input, and have been found with within the tissues of fish residing in those areas^1^,^9^.

Since FLX increases extracellular 5-HT levels, any physiological mechanism or behavior within an aquatic organism controlled by 5-HT may be susceptible to interference. One such mechanism is the response to low environmental dissolved oxygen, or hypoxia. Teleost fish periodically experience hypoxia, a common environmental phenomenon that is intensified by elevated water temperatures, reduced water exchange rates, high rates primary productivity or the discharge of municipal wastewater with high biological/chemical oxygen demand^10^,^11^. Hypoxia is thought to be sensed by specialized cells within the teleost fish gill, the neuroepithelial cells (NECs), found in all fish studied to date^12^,^13^,^14^. These cells are the homologues and evolutionary precursors of O_2_-sensing glomus cells of the mammalian carotid bodies^12^,^15^,^16^,^17^,^18^. NECs have been shown to depolarize in response to acute hypoxia^12^ and increase in size when hypoxia exposure is sustained^12^,^19^. Most NECs contain 5-HT^18^, which is thought to be released from the NECs in response to hypoxia^20^,^21^. It is currently unknown how NECs obtain 5-HT; however, mammalian glomus cells acquire 5-HT through a combination of 5-HT reuptake by SERT and synthesis of 5-HT *de novo* from endogenous tryptophan^22^. Interestingly, isolated perfused trout gills have been shown to extract 80% of the 5-HT from the perfusate^23^, which could indicate a high capacity for 5-HT reuptake.

After detecting hypoxia, teleost fish react with a suite of cardiovascular and ventilatory responses: a decrease in heart rate (fH) or bradycardia, a change in systemic and/or branchial vascular resistance resulting in changes in blood pressure, and an increase in ventilation amplitude (V_AMP_) and/or ventilation frequency (fV), all responses aimed to optimize O_2_ exchange across the gill and maintain metabolic rate^24^,^25^,^26^,^27^,^28^,^29^,^30^,^31^,^32^,^33^,^34^,^35^.

5-HT is a potent vasoconstrictor of the branchial and systemic vasculature; however, its role in the hypoxia response is not well understood^36^,^37^. Arterial injection of exogenous 5-HT results in an increase in gill vascular resistance that, in most cases, causes a counterintuitive decrease in O_2_ uptake across the gill^36^,^38^,^39^,^40^,^41^. This suggests that circulating 5-HT does not control hypoxia through vascular responses that promote O_2_ uptake and could, in fact, be detrimental to survival following hypoxic insults. 5-HT also modulates fH and V_AMP._ Consistent with the hypoxia response, arterial 5-HT injection stimulates hyperventilation^38^,^42^,^43^,^44^, but has variable effects on fH, resulting in both bradycardia^38^,^43^,^45^ and tachycardia^44^,^46^. As expected, intra-arterial (IA) injection of FLX has been recently shown to result in cardiovascular and ventilatory responses that are similar to those induced by 5-HT alone^44^,^47^.

Considering the involvement of 5-HT in the hypoxia response and that environmental hypoxia as a result of excessive primary productivity can be exacerbated by eutrophication through anthropogenic waste^10^,^11^, it is critical to establish whether the combination of FLX exposure and environmental hypoxia has detrimental effects on fish. We hypothesize that FLX treatment may attenuate or abolish the cardiovascular and/or ventilatory response to hypoxia, either by disrupting O_2_ sensing by fish NECs or by stimulating or desensitizing peripheral 5-HT receptors that mediate cardiovascular and ventilatory changes as a consequence of increased circulating 5-HT. To test this hypothesis, Gulf toadfish, *Opsanus beta*, were first injected IA with FLX to determine the cardiovascular and ventilatory effects of FLX alone. Toadfish were then treated intraperitoneally (IP) with FLX and exposed to hypoxia to determine whether FLX treatment interfered with the cardiovascular and ventilatory response to hypoxia.

## Results

### Intra-arterial (IA) FLX injection

**Figure 1:**
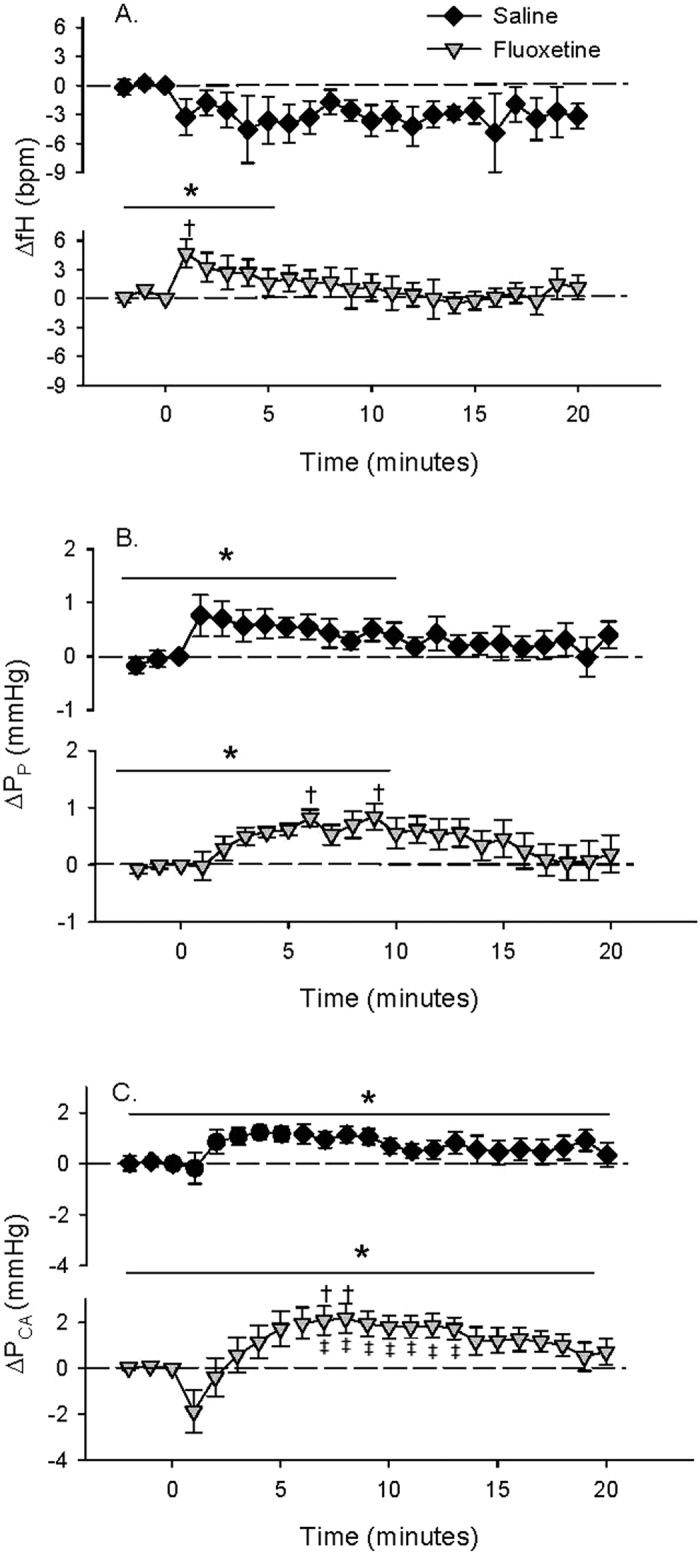
The effect of IA saline (1 μl saline^−1^.g fish^−1^; *N* = 7) or FLX (3.4 μg. μl saline^−1^.g fish^−1^; *N* = 9) injection on (**A**) fH, (**B**) P_P_ or (**C**) P_CA._ Injections occur at t = 0. Δ values are means ± S.E.M; *P < 0.05 indicates a significant change across the time points specified by the line as measured by one-way RM ANOVA; ^†^*P* < 0.05 indicates a significant difference from the value at t = 0; ^‡^*P* < 0.05 indicates a significant difference from the value at t = 1.

**Figure 2:**
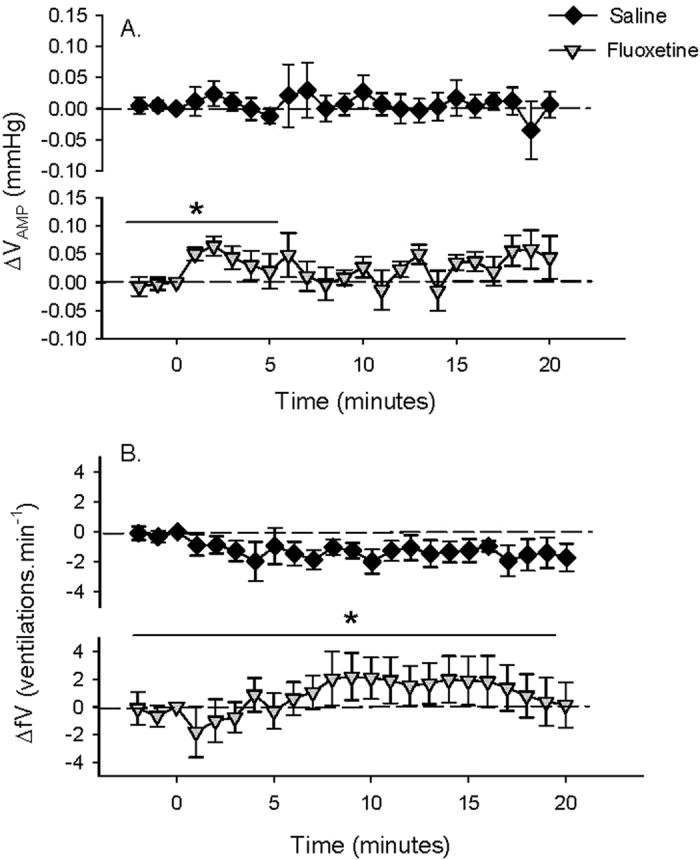
The effect of IA saline (1 μl saline^−1^.g fish^−1^; *N* = 7) or FLX (3.4 μg. μl saline^−1^.g fish^−1^; *N* = 9) injection on (**A**) V_AMP_ or (**B**) fV. Injections occur at t = 0. *P < 0.05 indicates a significant change across the time points specified by the line as measured by one-way RM ANOVA.

During the pre-injection period, there were no significant differences measured between the heart beat frequency (fH; beats·min^−1^), pulse pressure (P_P_; mmHg), which was used as an indicator of heart stroke volume, caudal arterial blood pressure (P_CA_; mmHg), ventilation amplitude (V_AMP_; mmHg), and ventilation frequency (fV; ventilations·min^−1^) of saline- and FLX-injected fish (Table 1). IA injection of FLX resulted in a pronounced but transient 14.0 ± 6.0 (8) % increase in fH compared to pre-injection values (Table 1) that was not measured in control fish injected with saline alone (Fig. 1A). Both saline and FLX injection resulted in a significant increase (52.1 ± 23.4 (7) % and 29.1 ± 7.2 (8) %, respectively) in P_P_ compared to pre-injection values (Table 1) over the first 10 minutes post-injection (Fig. 1B). Toadfish that were injected with FLX experienced a biphasic response that was not measured in saline-injected fish (Fig. 1C). Specifically, FLX-injected fish experienced a transient 12.0 ± 5.5 (9) % decrease in P_CA_ within the first minute post-injection compared to pre-injection values (Table 1 and Fig. 1C). P_CA_ in both saline- and FLX-injected fish then reached similar peak values within 5 minutes that were 10.6 ± 6.7 (7) % and 9.8 ± 3.1 (9) % higher, respectively, than pre-injection values (Table 1 and Fig. 1C). However, FLX-injection resulted in a 28.4 ± 8.5 (9) % increase in P_CA_ compared to values measured immediately post-injection (*t* = 1 min), compared to only a 10.7 ± 5.2 (7) % increase in saline-injected fish (Fig. 1C). FLX injection also resulted in a transient 19.3 ± 4.9 (8) % increase in V_AMP_ that was not measured in saline-injected fish (Fig. 2A). Furthermore, FLX injection resulted in a modest but significant increase in fV after injection that was not measured in saline-injected fish (Fig. 2B).

### Intraperitoneal (IP) FLX injection with hypoxia exposure

**Figure 3:**
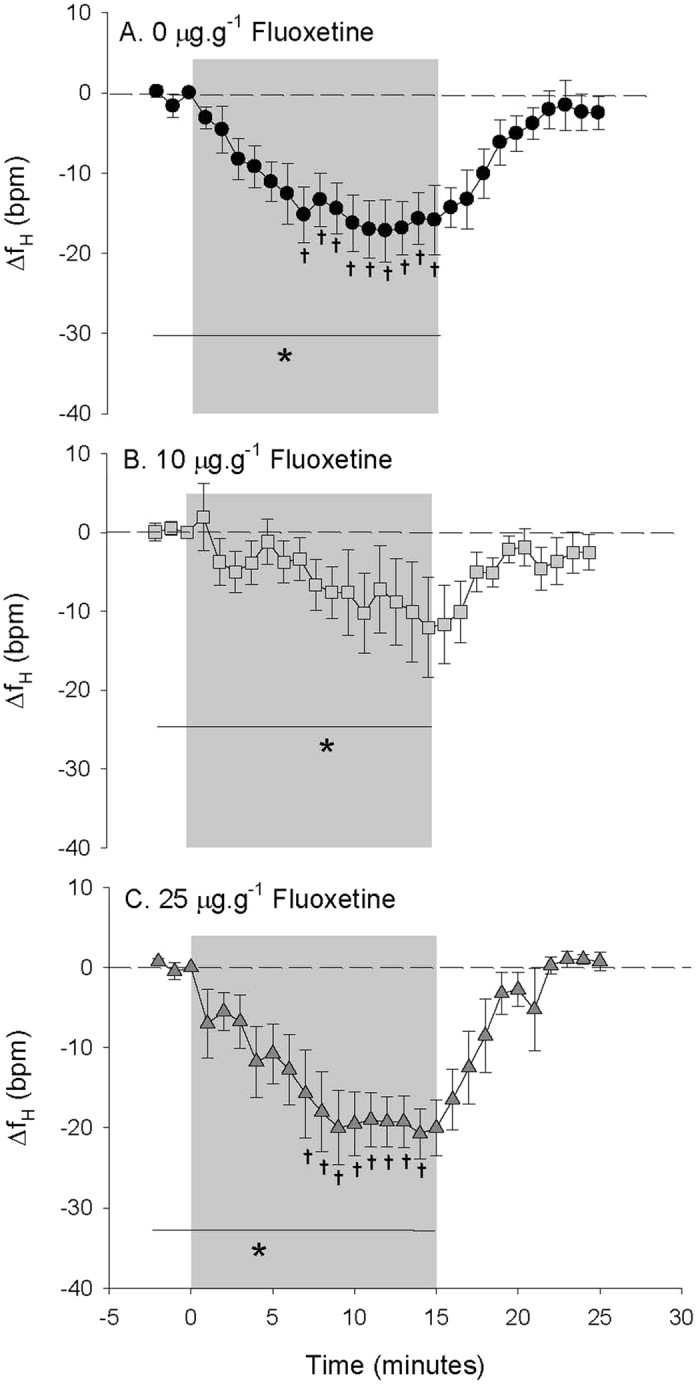
Heart rate (fH) in (**A**) fish treated with 0 μg FLX·g fish^−1^ (control; *N* = 11) (**B**) 10 μg FLX·g fish^−1^ (*N* = 9) or (**C**) 25 μg FLX·g fish^−1^(*N* = 6) during normoxia and in response to hypoxia exposure over time. Gray boxes indicate hypoxia exposure. Δ values are means ± S.E.M; *P < 0.05 indicates a significant change across the time points specified by the line as measured by one-way RM ANOVA; ^†^*P* < 0.05 indicates a significant difference from the value at t = 0.

**Figure 4:**
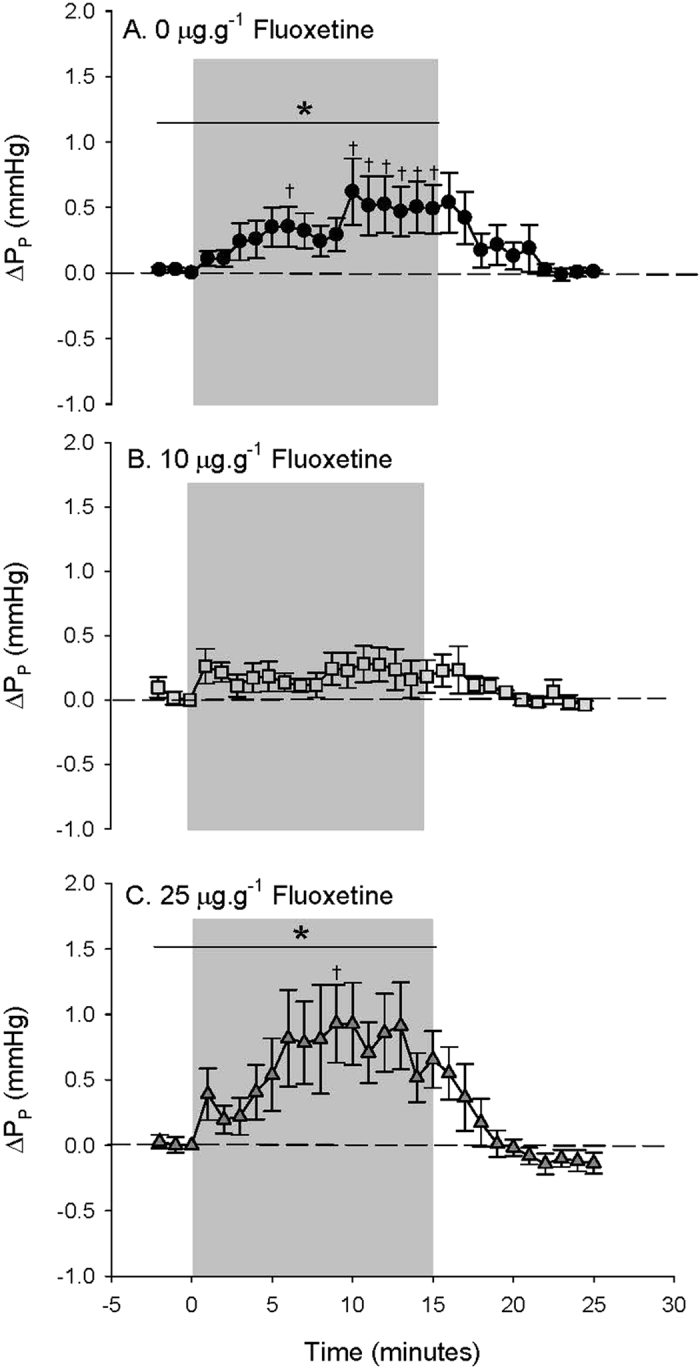
Pulse pressure (P_P_) in (**A**) fish treated with 0 μg FLX·g fish^−1^ (control; *N* = 8) (**B**) 10 μg FLX·g fish^−1^ (*N* = 4) or (**C**) 25 μg FLX·g fish^−1^ (*N* = 6) during normoxia and in response to hypoxia exposure over time. Δ values are means ± S.E.M; *P < 0.05 indicates a significant change across the time points specified by the line as measured by one-way RM ANOVA; ^†^*P* < 0.05 indicates a significant difference from the value at t = 0.

**Figure 5:**
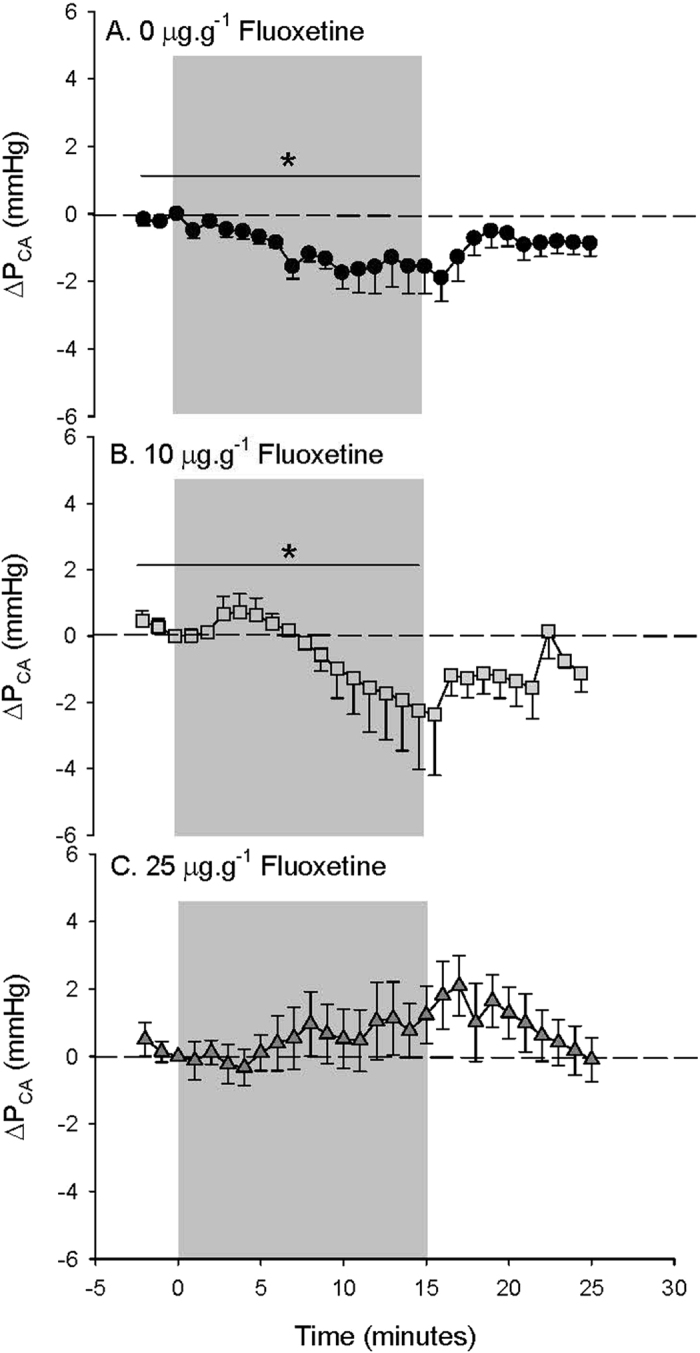
Caudal arterial blood pressure (P_CA_) in (**A**) fish treated with 0 μg FLX·g fish^−1^ (control; *N* = 8) (**B**) 10 μg FLX·g fish^−1^ (*N* = 4) or (**C**) 25 μg FLX·g fish^−1^ (*N* = 6) during normoxia and in response to hypoxia exposure over time. Δ values are means ± S.E.M; *P < 0.05 indicates a significant change across the time points specified by the line as measured by one-way RM ANOVA.

Circulating 5-HT concentrations 24 h after IP injection were not significantly different between control (6.1 ± 0.5 (7) ng.ml^−1^) and 10 μg.g^−1^ FLX-treated fish (6.2 ± 0.7 (7) ng.ml^−1^). Due to technical difficulties, plasma 5-HT concentrations were not measured in 25 μg.g^−1^ FLX-treated fish. During the initial normoxia period, there were no significant differences measured between the cardiovascular parameters of control and FLX-treated fish (Table 2). When exposed to hypoxia (~0.5–1.0 mg·L^−1^ dissolved oxygen), control toadfish exhibited significant bradycardia, with a significant 38.0 ± 5.0 (11) % reduction in fH compared to normoxia values (Table 2 and Fig. 3A). Fish treated with 10 μg·g^−1^ FLX also showed a significant bradycardia in response to hypoxia over time (Fig. 3B); however, the response was highly variable and attenuated compared to controls (only a 17.4 ± 12.5 (9) % reduction compared to normoxia values; Table 2). In contrast, fish treated with the higher 25 μg·g^−1^ dose of FLX had a pronounced bradycardia similar to that of the control fish (38.2 ± 5.1 (6) % reduction compared to normoxia values (Table 2); Fig. 3C *cf.* Fig. 3A). Upon recovery from hypoxia exposure, fH returned to values measured during normoxia for all treatment groups (Fig. 3). Furthermore, control toadfish responded to hypoxia with a significant 47.8 ± 12.0 (8) % increase in P_P_ compared to normoxia values (Table 2) that was not measured in fish treated with 10 μg·g^−1^ FLX (Fig. 4A *cf.* Fig. 4B). However, in fish treated with 25 μg·g^−1^ FLX, the P_P_ response was similar to response in control fish, with a significant 68.7 ± 14.6 (6) % increase compared to values measured during normoxia (Table 2 and Fig. 4C). Upon recovery from hypoxia exposure, P_P_ returned to values measured during normoxia in both control and 25 μg·g^−1^ treated fish (Fig. 4). Control toadfish responded to hypoxia with a subtle but significant 6.4 ± 1.8 (8) % decrease in P_CA_ compared to values measured during normoxia (Table 2) that reached a minimum (8.9 ± 3.7 (8) % lower P_CA_ compared to values measured during normoxia) during the last 5 minutes of hypoxia exposure (Fig. 5A). Fish treated with 10 μg·g^−1^ FLX showed a biphasic response with an initial transient and slight increase in P_CA_ followed by a decrease in P_CA_ that reached a minimum (7.0 ± 4.8 (4) % lower than values measured during normoxia; Table 2) during the last 5 minutes of hypoxia exposure comparable to controls (Fig. 5B). In contrast, 25 μg·g^−1^ FLX-treated fish showed no significant P_CA_ response to hypoxia (Fig. 5C). Upon recovery from hypoxia exposure, P_CA_ returned to values measured during normoxia in both control and 10 μg·g^−1^ FLX-treated fish (Fig. 5).

**Figure 6:**
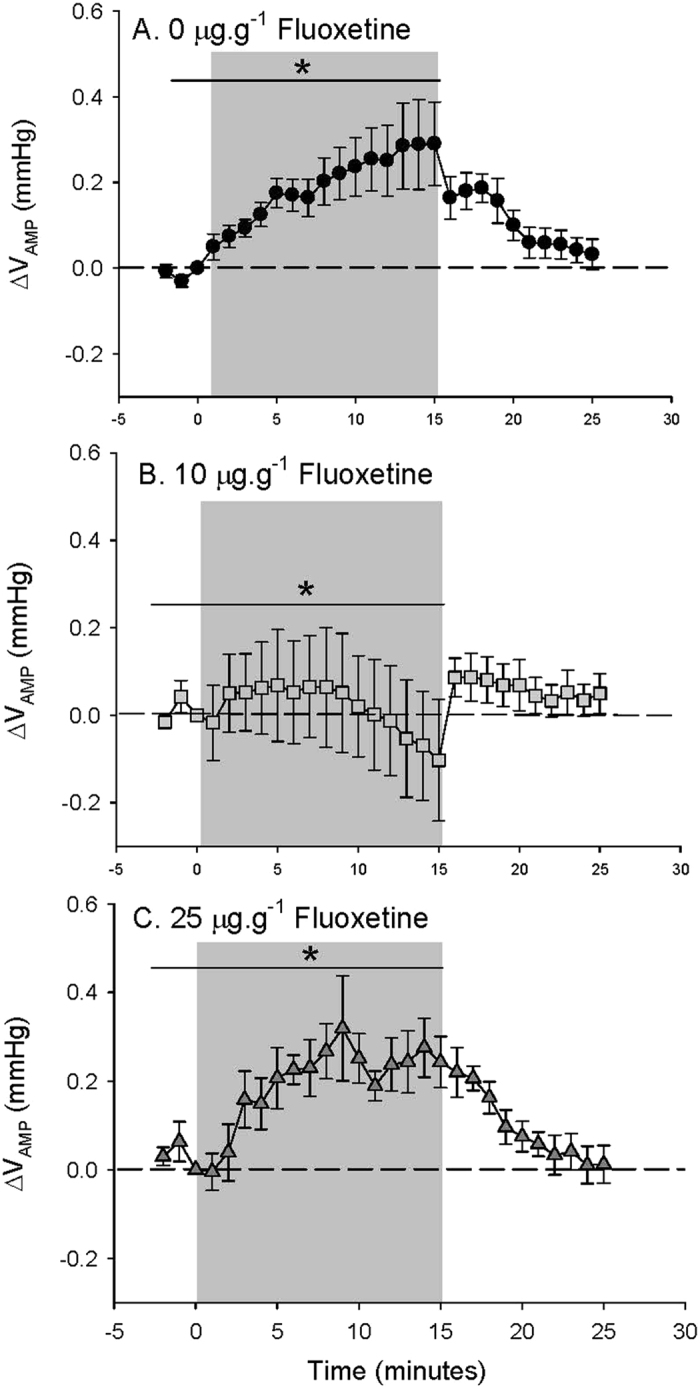
Ventilatory amplitude (V_AMP_) in (**A**) fish treated with 0 μg FLX·g fish^−1^ (control; *N* = 12) (**B**) 10 μg FLX·g fish^−1^ (*N* = 5) or (**C**) 25 μg FLX·g fish^−1^ (*N* = 5) during normoxia and in response to hypoxia exposure over time. Δ values are means ± S.E.M; *P < 0.05 indicates a significant change across the time points specified by the line as measured by one-way RM ANOVA.

During the initial period of normoxia, there were no significant differences measured between the ventilatory parameters of control and FLX-treated fish (Table 2). Control toadfish responded to hypoxia with an increase in V_AMP_ that reached a maximum 86.6 ± 17.2 (12) % during the last 5 min of hypoxia exposure compared to values measured during normoxia (Table 2 and Fig. 6A). In contrast, the V_AMP_ response of 10 μg·g^−1^ FLX-treated fish to hypoxia was highly variable (a 10.3 ± 22.5 (5) % increase during the last 5 min of hypoxia exposure compared to values measured during normoxia; Table 2 and Fig. 6B) and less pronounced than control toadfish. Fish treated with 25 μg·g^−1^ FLX experienced a 44.1 ± 13.7 (6) % increase in V_AMP_ during the last 5 min of hypoxia compared to values measured during normoxia (Table 2 and Fig. 6C). Upon recovery from hypoxia exposure, V_AMP_ returned to values measured during normoxia in all treatments (Fig. 6). There were no significant changes in fV in response to hypoxia in control fish or either treatment group (data not shown).

## Discussion

The hypothesis of the present study was that SERT inhibition by FLX results in elevated extracellular 5-HT levels and attenuates or abolishes the cardiovascular and/or ventilatory response to hypoxia, either by disrupting O_2_ sensing by fish NECs or by stimulating and potentially desensitizing peripheral and/or central 5-HT receptors. These changes were hypothesized to mediate changes in fH, vascular resistance, blood pressure, V_AMP_ and/or fV. Toadfish treated with two different IP doses of FLX showed differential responses to hypoxia. Fish treated with 10 μg.g^−1^ FLX showed a slight attenuation in their fH, P_P_ and V_AMP_ responses during hypoxia, while those treated with 25 μg.g^−1^ did not show this effect. However, there was a disruption of the P_CA_ response with the low dose of FLX that was eliminated in fish treated with 25 μg.g^−1^ FLX. There was no effect of hypoxia or FLX treatment on fV. Because FLX treatment impacted each cardiovascular and ventilatory response to hypoxia differently, we believe that interference with the hypoxia response is most likely at the level of peripheral and/or central 5-HT receptors that control these responses individually and not at O_2_ sensation in these fish; however future work should directly investigate the NEC response to FLX exposure.

To date, only one other study has investigated the impact of FLX on the cardiovascular and ventilatory responses of fish^47^. Therefore, the following discussion focuses on both the findings of that study as well as the known effects of 5-HT alone, since FLX treatment, in inhibiting SERT, increases extracellular 5-HT. Toadfish injected IA with FLX experienced a significant tachycardia as well as a biphasic P_CA_ response characterized by a transient decrease in P_CA_ followed by a prolonged elevation. These findings suggest the potential for FLX treatment to reduce survival during hypoxia exposure as both tachycardia and an elevation in P_CA_ are in direct conflict with the typical cardiovascular response to hypoxia of toadfish, which includes a significant bradycardia and a decrease in P_CA_^35^ (see below).

The tachycardia experienced by FLX-injected toadfish is consistent with previous findings in rainbow trout injected with either FLX^47^ or 5-HT^44^,^46^. In mammals, 5-HT-induced tachycardia is likely mediated by the 5-HT_4_ receptor^48^ and while its presence in teleost heart has not yet been investigated, the 5-HT_2A_ receptor is expressed at low levels providing evidence for direct serotonergic control of the teleost heart^49^. Furthermore, the highest amount of SERT transcript within toadfish has been found in the heart (M.H.B. Amador and M.D. McDonald, unpublished) and 5-HT has been measured in the ventricle of the European conger^50^. Similar to mammals, the role of 5-HT in the regulation of fH in fish is complex as injection of 5-HT or the 5-HT_2_ agonist, α-methyl-5-HT has also been demonstrated to cause bradycardia^35^,^38^,^43^,^45^. In toadfish, the bradycardia response to α-methyl-5-HT was inhibited by the 5-HT_2A_ receptor antagonist, ketanserin, suggesting the involvement of 5-HT_2A_ receptors^35^,^49^. While the 5-HT_2A_ receptor has been shown to be expressed in the heart and may directly mediate these effects, the 5-HT-induced bradycardia may also be indirect, as evidence in both fish and mammals suggests that it could also be a consequence of a vagal stimulation mediated by central 5-HT receptors^43^,^48^. In the present study, toadfish injected with FLX also experienced an increase in P_P_ , which is an indicator of cardiac stroke volume (SV). The increase in both fH and P_P_ signifies that cardiac output (CO = fH × SV) was elevated in FLX-injected fish. This is in contrast to the study by Janvier *et al.*^43^, in which eel experienced a decrease in CO, but also a decrease in fH or bradycardia, in response to 5-HT injection.

Taking the above into account, the elevation in P_CA_ measured in FLX-injected fish could be due, in part, to the FLX-induced increase in CO. However, 5-HT is a well-described vasoconstrictor of the vertebrate branchial/pulmonary and systemic vasculature^36^,^38^,^39^,^45^,^48^,^51^,^52^,^53^. Consistent with the increase in P_CA_ measured in toadfish, FLX and 5-HT injection in rainbow trout cause a similar increase in systemic blood pressure, as measured by an increase in dorsal aortic blood pressure (P_DA_)^44^,^47^. Furthermore, in response to α-methyl 5-HT, there are increases in P_CA_ and P_DA_ that occur with a simultaneous decrease in fH in toadfish and no change in fH in the trout^35^,^44^. Unlike fH, this response to α-methyl 5-HT was insensitive to ketanserin in toadfish, suggesting the involvement of 5-HT_2B/2C_ receptors and further dissociating the P_CA_ response from the fH response^35^. However, decreases in P_DA_ in response to 5-HT injection have also been shown in the rainbow trout, eel and the Antarctic borch and, indeed in the present study, a transient decrease in P_CA_ was observed within the first minute of FLX injection. A decrease in systemic blood pressure is believed to be due to an intense branchial vasoconstriction without the simultaneous increase in systemic vasoconstriction^38^,^43^,^45^,^46^. The biphasic response in toadfish suggests that branchial vasoconstriction, which would register as a decrease in P_CA_ downstream from the gill, occurs first and then is followed by a systemic vasoconstriction, registering as an increase in P_CA_. The adaptive significance of a FLX- (or 5-HT-) induced increase in systemic vascular resistance has not been directly investigated but illustrates the potential importance of SERT in preventing uncontrolled systemic vasoconstriction in response to 5-HT.

Toadfish experienced a subtle hyperventilatory response to FLX, due to a transient increase in V_AMP_ and a slight but prolonged elevation in fV. This is in contrast to a recent study on trout wherein a hyperventilatory response was not elicited by IA-FLX or 5-HT injection, only intracerebroventricular (ICV) injection, suggesting that the effect may be centrally mediated^44^,^47^. The different responses could be due to the fact that toadfish in the present study were injected with 17-times higher concentrations of FLX than trout, which may have had central as well as systemic effects. That being said, the effect of FLX on toadfish fV was similar to that in trout and eel injected IA with 5-HT^43^,^46^. Furthermore, both toadfish and trout V_AMP_ and fV increase in response to α-methyl 5-HT administered IA and ICV, respectively^35^,^44^, indicating that in both species, ventilation is controlled, at least in part, by 5-HT via the 5-HT_2_ receptor family. In the case of toadfish, the V_AMP_ response was attenuated and the fV response completely inhibited by ketanserin, supporting a dissociation between the two responses that is also apparent in the present study^35^.

### Intraperitoneal (IP) FLX injection with hypoxia exposure

Both control and IP-FLX injected fish in the present study had fH, P_P_ , P_CA_, V_AMP_ and fV values during normoxia that were not significantly different from each other, illustrating that IP FLX treatment did not affect resting cardiovascular or ventilatory parameters. It was not until fish were challenged with hypoxia that differences between control and FLX-treated fish became apparent. That IP-FLX treatment, which was 3–7 times higher that IA-FLX treatment, had no effect during normoxia was likely a reflection of the slow release of FLX into the intraperitoneal cavity and its subsequent absorption, which may have resulted in a lower circulating dose of FLX and a less direct action on the systemic vasculature than IA injection. The cardiovascular and ventilatory parameters during normoxia measured in the present study were slightly lower than those measured in toadfish in a previous study^35^, most likely due to longer post-surgery recovery times in the present study (36–48 h compared to 24 h).

In control toadfish, hypoxia exposure resulted in the typical suite of cardiovascular and ventilatory responses that are relatively consistent across fish species^24^,^31^. Control toadfish experienced a significant bradycardia that was matched by an increase in P_P_, suggesting that CO was likely maintained. In these fish, P_CA_ significantly decreased and, given the constant CO, this was most likely a consequence of branchial vasoconstriction as a way to increase lamellar perfusion and optimize oxygen uptake^36^,^41^ without a simultaneous increase in systemic vasoconstriction. Similar to a study by McDonald and coworkers^35^, toadfish responded with an increase in V_AMP_ without a significant change in fV.

Given that IA-FLX injection resulted in tachycardia, it might be expected that IP-FLX treatment would interfere with the decrease in fH typically observed in response to hypoxia. As anticipated, a slight attenuation and higher variability in the bradycardia response was measured in toadfish pre-treated with the lower 10 μg.g^−1^ FLX dose. However, the full bradycardia response was present in fish exposed to the higher 25 μg.g^−1^ FLX dose. The same tendency for attenuation at the lower FLX dose and with no effect at the higher dose was evident in both the P_P_ and V_AMP_ response to hypoxia. These results may be a factor of circulating 5-HT concentrations and 5-HT receptor populations. In the present study, circulating 5-HT concentrations in the 10 μg.g^−1^ FLX-treated fish were not significantly different than the control fish, suggesting that the increase in extracellular 5-HT due to FLX-induced inhibition of SERT impacted only adjacent 5-HT receptors (*e.g.,* synaptic or paracrine signaling). Unfortunately, in the present study, circulating 5-HT concentrations were not measured in the 25 μg.g^−1^ FLX-treated fish; however, previous studies have shown that this dose of FLX does result in a significant increase in circulating 5-HT concentrations^2^,^3^. This more pronounced, widespread increase in 5-HT (*e.g.,* humoral signaling) might have affected both high and low affinity 5-HT receptor sub-types distributed throughout the body. If the function or the conditions under which the various receptor populations desensitize differ, the effect of FLX on the overall cardiovascular or ventilatory response could also vary dramatically^48^,^54^.

IA-FLX injection also resulted in biphasic change P_CA_, a transient decrease followed by a more prolonged increase that was in direct conflict with the typical toadfish hypoxia response, leading to the prediction that IP-FLX treatment would interfere with the prolonged decrease in P_CA_ observed in response to hypoxia. Indeed, IP-FLX treatment caused a dose-dependent disturbance in the P_CA_ response to hypoxia: it was attenuated at 10 μg.g^−1^ FLX and completely abolished at 25 μg.g^−1^ FLX. Thus, IP treatment of FLX disrupts the natural P_CA_ response to hypoxia, a response that may be vital to efficient O_2_ uptake across the gill in times of limited environmental O_2_. The mechanism by which this occurs is not entirely clear, partly because of the confounding role of 5-HT in the control of gill blood flow during hypoxia^36^,38–40. However, our previous work has shown that the decrease in P_CA_ in toadfish in response to hypoxia or the O_2_-chemoreceptor stimulant, NaCN, is attenuated and significantly reduced, respectively, by the 5-HT_1/2_ receptor antagonist, methysergide, suggesting a role for 5-HT in the hypoxia response of these fish^35^. More specifically, in toadfish 5-HT-induced changes in both systemic and branchial vascular resistance are believed to be mediated by the 5-HT_2B/2C_ receptor based on sensitivity to α-methyl 5-HT but insensitivity to ketanserin^35^ and, in the case of branchial vasoconstriction, further inhibited by the 5-HT_2B/2C_ receptor antagonist, SB206553 (M.D. McDonald, unpublished). Potentially, the chronic increase in extracellular 5-HT associated with FLX-induced inhibition of SERT led to a desensitization of the 5-HT receptors, potentially 5-HT_2B/2C_ receptors, that are at least partly responsible for the P_CA_ response in toadfish. Furthermore, an increase in circulating 5-HT, which is likely occurring in fish treated with 25 μg.g^−1^ FLX^2^,^3^ has been shown to result in an increase in branchial vascular resistance but a decrease in O_2_ uptake across the gill that could be detrimental to fish when facing hypoxia^36^,^38^,^40^. Indeed, injection of only 0.1–0.2 μg.g^−1^ 5-HT has been shown to induce branchial vasoconstriction, oppose lamellar recruitment and reduce arterial PO_2_ in trout gill preparations^38^,^39^. While resting P_CA_ in FLX-treated fish was not lower than controls, implying that branchial vascular resistance was not chronically elevated in these fish, it cannot be ruled out that IP-FLX treatment resulted in a chronic increase in both branchial and systemic (as measured in response to IA-FLX injection) vascular resistance, two opposing effects that would not result in a detectable change in P_CA_ but would result in a reduction in arterial PO_2_.

The data of the present study suggest that when SERT are inhibited by pharmacological doses of FLX there are potentially negative consequences to survival in low oxygen environments, the most apparent being the elimination of the P_CA_ response to hypoxia that, while modest, could reflect a substantial adjustment in the branchial circulation and reduction in arterial PO_2_. Whether the disruption in the hypoxia response that occurs in response to acute, pharmacological doses of FLX (IP dose of 10 and 25 μg.g^−1^) administered in the present study are reflective of what might occur in response to a chronic exposure to environmentally realistic FLX concentrations (waterborne dose of 0.01 μg.L^−1^ and approximate body burden of 0.001 μg.g^−1^)^7^,^9^ is difficult to predict, especially considering the atypical dose response measured in the present study. However, if the present findings are indicative of what may occur in response to exposure to environmentally realistic FLX concentrations in combination with naturally occurring hypoxia events; a possible scenario within the Gulf of Mexico, known for its hypoxic “dead-zones” and anthropogenic waste input, there could be profound implications. This may be especially true for aquatic organisms that are particularly sensitive to hypoxia, for which subtle changes in the ability to maintain arterial PO_2_, which would occur if the bradycardic, hyperventilatory or vascular responses to hypoxia were disrupted, would lead to a reduction in survival. This research emphasizes the importance of studies that investigate toxicity in combination with other environmental challenges; perhaps especially true in the case of chronic, sub-lethal toxicant exposure, when resting conditions of organisms may not be affected but effects become evident once the organism is challenged. Future work needs to further address the role of SERT in the control of circulating 5-HT and the regulation of vascular resistance in fish. Future work will also investigate whether environmentally relevant doses of waterborne FLX, its potent metabolite norfluoxetine, or other SSRIs will have a negative impact on marine organisms when exposed to hypoxic environments.

## Materials and Methods

### Experimental Animal

Gulf toadfish, *Opsanus beta,* were captured by commercial shrimpers using roller trawls in Biscayne Bay, Florida (Florida Fish and Wildlife Conservation Commission Special Activity License #SAL-12-0729-SR) and immediately transferred to the laboratory where they were held for up to one month in 50-L glass aquaria with flowing, aerated seawater at a temperature of 20–24 °C and a density of 10 g fish·L^−1^. Fish were treated with a dose of malachite green (final concentration 0.05 mg·L^−1^) in formalin (15 mg·L^−1^) (AquaVet) on the day of transfer to the laboratory to prevent infection by the ciliate, *Cryptocaryon irritans*^55^. Fish were fed weekly with thawed squid. Procedures described in this study were approved by the University of Miami Animal Care and Use Committee (IACUC) in accordance with the Office of Laboratory Animal Welfare (OLAW) National Institutes of Health (assurance #A-3224–01) and the Council on Accreditation of the Association for Assessment and Accreditation of Laboratory Animal Care (AAALAC International).

### Experimental Procedures

#### Intra-arterial (IA) FLX injection

The first set of experiments investigated the effect of IA FLX (fluoxetine-HCl, Sigma-Aldrich) injection, which targets transporters responsible for 5-HT reuptake (SERT), on cardiovascular and ventilatory parameters: heart beat frequency (fH; beats·min^−1^), pulse pressure (P_P_; mmHg), which was used as an indicator of heart stroke volume, caudal arterial blood pressure (P_CA_; mmHg), ventilation amplitude (V_AMP_; mmHg), and ventilation frequency (fV; ventilations·min^−1^). Toadfish were surgically implanted with caudal arterial catheters (Clay Adams PE 50 tubing; Becton Dixon) that were inserted into the caudal artery, with a modified approach originally used by Wood *et al.*^56^ and McDonald *et al.*^57^. Specifically, a small, 1 cm incision was made along the right-hand side of the tail to expose the caudal artery. Then, using a sharpened guitar string threaded inside a 30 cm length of PE 50 tubing, the cannula was carefully fed into the caudal artery. Because the guitar wire exceeded the length of the PE tubing by a millimeter, the artery was punctured before the cannula was introduced, facilitating appropriate placement of the cannula into the artery, rather than the vein. The catheter was filled with a heparinized (Sigma-Aldrich; 50 i.u.·ml^−1^) 150 mmol·L^−1^ NaCl saline solution. The catheter was secured and the wound treated with oxytetracycline (Sigma-Aldrich) and sutured closed as described previously^56^,^57^. This caudal arterial catheter served as the injection site for the FLX and allowed for the measurement of fH, P_CA_ and P_P_ (see below). For the measurement of V_AMP_ and fV, opercular catheters (Clay Adams PE 160 tubing; Becton Dixon) were surgically inserted through the opercular epithelium of the fish so that the open end of the catheter rested inside the opercular cavity as described previously^35^. After surgery, fish were left to recover for 36–48 h in 1.5 L shielded, chambers that were supplied with aeration to maintain dissolved oxygen levels at 100% air saturation and clean, flowing seawater. Post recovery, pressure transducers connected to an amplifier and data acquisition system (MP150; Biopac Systems) were attached to the caudal arterial and opercular catheters. These pressure transducers were calibrated before each experiment using a static water column. Resting cardiovascular and ventilatory parameters were monitored for at least 20 min before FLX (3.4 μg. μl saline^−1^.g fish^−1^, *N* = 9; (mean weight: 0.110 ± 0.022 kg) or saline alone (1 μl saline^−1^.g fish^−1^, *N* = 7; mean weight: 0.142 ± 0.021 kg) was injected into the caudal arterial catheter followed by 3 μl saline^−1^.g fish^−1^ to ensure complete drug delivery into the circulation. Cardiovascular and ventilatory parameters were monitored for another 20 min post-injection.

#### Intraperitoneal (IP) FLX injection with hypoxia exposure

A second set of experiments investigated the impact of IP FLX treatment on the ability of toadfish to mount a cardiovascular and ventilatory response to environmental hypoxia (dissolved oxygen concentrations ~0.5–1.0 mg.L^−1^). The IP injection of compounds like fluoxetine in oil vehicles (*e.g.*, peanut oil, coconut oil, corn oil) mediates the slow release of these substances into the circulation^2^. Toadfish were surgically implanted with caudal arterial and opercular catheters as described above. After surgery, toadfish were either treated with 0 μg FLX·5 μL coconut oil^−1^·g fish^−1^ (controls: 0.100 ± 0.008 kg, *N* = 13), 10 μg FLX·5 μL coconut oil^−1^·g fish^−1^; (0.100 ± 0.010 kg. *N* = 9) or 25 μg FLX·5 μL coconut oil^−1^·g fish^−1^ (0.116 ± 0.010 kg, *N* = 7) via intraperitoneal injections with a 500 μL Hamilton leur lock syringe (Hamilton Company) attached to a disposable 18 gauge needle. After injection, the fish were placed on ice for approximately 15 seconds to solidify the coconut oil implant as previously described^2^. After surgery and treatment, toadfish were allowed to recover as described above.

After recovery, blood samples (200 μl) were taken from the caudal arterial catheter with a 500 μL Hamilton syringe, centrifuged for 10 minutes at 16,000 rpm, the plasma decanted, frozen in liquid N_2_ and stored at −80 °C until analyzed for 5-HT concentrations. Pressure transducers were then connected to the caudal arterial and opercular catheters to measure fH, P_P_, P_CA_, V_AMP_ and fV during normoxia and hypoxia in fish of the three treatment groups. The toadfish were exposed to a 10-min interval of normoxia (~7–8 mg·L^−1^ dissolved oxygen), followed by a 15-min interval of hypoxia (~0.5–1.0 mg·L^−1^ dissolved oxygen). Hypoxic conditions were administered by replacing the normal air supply with either a 2.5% O_2_ balance N_2_ mixture (Airgas) or 100% N_2_ gas (Airgas); the minimum dissolved oxygen concentration was monitored and did not differ between the two methods. After the 15-min hypoxia interval, the gas was switched back to air for a recovery period of 10 min resulting in a total experiment length of 35 min.

### Analytical Techniques

Data for P_CA_, P_P_ and V_AMP_ obtained from the BioPac MP150 system were processed using the acquisition software AcqKnowledge 4.1. Every minute, 5-sec trace segments, free of artifact (due to fish movement, hiccups etc…), were selected for the measurement of P_CA_, P_P_ and V_AMP_ . Every minute, fH and fV were determined manually by counting the amount of time required for 10 beats and extrapolating that value to beats minute^−1^. In a few cases, individual traces were deemed unusable for certain fish because of low signal: noise ratio (fH, fV) or exceedingly high variability or instability (P_CA_, P_P_ ,V_AMP_) that made it impossible to find artifact-free data. Plasma 5-HT concentrations were analyzed using a 5-HT ELISA kit (Alpco Diagnostics).

### Statistical Analysis

Figures display Δ values (the value measured at *t* = 0, *i.e.*, immediately before injection or hypoxia, was subtracted from all other values). These and all other values were expressed as means ± the standard error of the mean (N). Statistical analysis was performed using the statistical package found in SigmaPlot 11.0 (Systat Software, Inc.). A Student’s unpaired t-test was used for examining differences in mean pre-injection or normoxia data between treatment groups. Differences over time within a treatment group was analyzed using either a one-way RM ANOVA or one-way ANOVA followed by a Holm-Sidak multiple comparisons test. Data that was not normally distributed was log transformed. If not normally distributed upon log transformation, a one-way RM ANOVA based on ranks or a one-way ANOVA based on ranks with time as the main factor followed by a Tukey’s multiple comparisons test was used.

## Additional Information

**How to cite this article**: Panlilio, J. M. *et al.* Treatment with the selective serotonin reuptake inhibitor, fluoxetine, attenuates the fish hypoxia response. *Sci. Rep.* **6**, 31148; doi: 10.1038/srep31148 (2016).

## Figures and Tables

**Table 1:** Pre-injection cardiovascular and ventilatory values of saline and IA FLX-injected fish.

	Saline	FLX
fH (BPM)	39.3 ± 2.8 (7)	37.2 ± 1.9 (8)
P_P_ (mmHg)	2.3 ± 0.6 (7)	2.2 ± 0.4 (9)
P_CA_ (mmHg)	14.6 ± 2.4 (7)	14.9 ± 1.5 (9)
V_AMP_ (mmHg)	0.34 ± 0.05 (7)	0.33 ± 0.06 (9)
fV (VPM)	20.7 ± 1.1 (7)	22.1 ± 1.1 (9)

All means ± SEM (N) are an average of measurements taken three minutes prior to injection.

**Table 2:** Normoxia cardiovascular and ventilatory values of control and IP FLX-treated fish.

	Control	10 μg·g fish^−1^	25 μg·g fish^−1^
fH (BPM)	40.3 ± 3.8 (11)	33.7 ± 4.9 (9)	50.3 ± 3.5 (6)
P_P_ (mmHg)	0.70 ± 0.28 (8)	0.54 ± 0.16 (4)	0.74 ± 0.18 (6)
P_CA_ (mmHg)	17.8 ± 2.1 (8)	21.5 ± 3.8 (4)	19.1 ± 2.5 (6)
V_AMP_ (mmHg)	0.30 ± 0.03 (12)	0.39 ± 0.14 (5)	0.42 ± 0.07 (5)
fV (VPM)	24.6 ± 1.9 (13)	25.0 ± 2.7 (6)	26.5 ± 2.8 (7)

All means ± SEM (N) are an average of measurements taken during the last three minutes of normoxia.
